# Differential access to psoriasis biologic information in English and Spanish

**DOI:** 10.1016/j.jdin.2023.12.001

**Published:** 2024-01-28

**Authors:** Max Oscherwitz, Katie Lovell, Dane Markham, Rita Pichardo, Steven R. Feldman

**Affiliations:** a Center for Dermatology Research, Department of Dermatology, Wake Forest School of Medicine, Winston-Salem, North Carolina; b Mayo Clinic Alix School of Medicine, Jacksonville, Florida; c Department of Pathology, Wake Forest School of Medicine, Winston-Salem, North Carolina; d Department of Social Sciences and Health Policy, Wake Forest School of Medicine, Winston-Salem, North Carolina; e Department of Dermatology, University of Southern Denmark, Odense, Denmark

**Keywords:** biologics, education, equity, psoriasis, Spanish, websites

*To the Editor:* Education is crucial in providing psoriasis patients with a comprehensive understanding of their disease and improving long-term management outcomes. Websites are becoming a valuable tool for delivering clinical psoriasis information. However, language can be a barrier to comprehension, particularly for non-English speakers in the United States. According to the US Department of Health and Human Services, roughly 28.4% of the 62.1 million LatinX individuals residing in the United States are not fluent in English.

Although LatinX populations within the United States have a lower prevalence of psoriasis compared with other ethnic groups, they are more likely to present with extensive disease, are disproportionately represented in clinical trials, and have a worse quality of life from the disease compared with Caucasian populations.^1^^,^^2^ LatinX patients with psoriasis are also more likely than Caucasian patients to receive biologics for treatment.^2^ We examined the English and Spanish versions of websites for psoriasis biologic medications to measure their quality as an educational resource.

Twelve biologic drugs are approved by the United States Food and Drug Administration to treat psoriasis and/or psoriatic arthritis. We created a quantitative metric to analyze these drugs’ corresponding English and Spanish websites. The metric examined the websites’ language-specific educational materials, resources, and supplemental materials.

Of the biologics with Spanish websites, 58.3% had additional links in Spanish on their homepage; 25% of these links led to the prescribing information translated into Spanish, and 33.3% led to Spanish-specific webpages with additional information (Tables I and II). When compared with English psoriasis biologic websites (100%), only 25% of Spanish psoriasis biologic websites included a description of psoriasis and payment resources. Descriptions of psoriasis subtypes (83%) and safety information, visual aids, contraindications/special populations, and links to the United States Food and Drug Administration adverse event reporting were found on 75% of Spanish psoriasis biologic websites versus 100% of English psoriasis biologic websites. English psoriasis biologic websites featured testimonials 92% of the time, compared with only 17% of Spanish psoriasis biologic websites.

**Table I:** Spanish biologic website analysis

Biologic	Spanish-specific link on biologic website homepage?	Spanish-specific description of psoriasis?	Psoriasis subtypes mentioned in Spanish?	Payment resources listed in Spanish?	Spanish-specific resources listed? (ie, information to contact Spanish-speaking representatives)	Safety information listed in Spanish? (ie, allergy information)	Visual aids present on website?	Testimonials present in Spanish?	Mention of contraindications and special populations in Spanish?	FDA information listed to report adverse medication events in Spanish?
Certolizumab pegol	No	No	Yes	No	Yes, phone services	Yes	Yes	No	Yes	Yes
Secukinumab	Yes∗	No	Yes	No	Yes, phone services	No	Yes	Yes	No	No
Etanercept	Yes (link broken)	Yes	Yes	Yes	Yes, phone services	Yes	Yes	No	Yes	Yes
Adalimumab	Yes†	No	Yes	No	No	Yes	Yes	No	Yes	Yes
Tildrakizumab-asmn	Yes∗	Yes	Yes	Yes	No	Yes	Yes	Yes	Yes	Yes
Infliximab	No	No	Yes	No	No	Yes	No	No	Yes	Yes
Brodalumab	No	No	No	No	No	No	No	No	No	No
Golimumab	Yes†	No	Yes	No	No	Yes	Yes	No	Yes	Yes
Risankizumab	No	No	No	No	Yes, phone services	No	Yes	No	No	No
Ustekinumab	Yes∗	Yes	Yes	Yes	Yes, phone services	Yes	Yes	No	Yes	Yes
Ixekizumab	Yes∗	No	Yes	No	No	Yes	Yes	No	Yes	Yes
Guselkumab	Yes†	No	Yes	No	No	Yes	Yes	No	Yes	Yes
Summary	Yes∗ 4/12 (33%), yes† 3/12 (25%), no 4/12 (33.3%)	Yes 3/12 (25%), no 9/12 (75%)	Yes 10/12 (83.3%), no 2/12 (16.7%)	Yes 3/12 (25%), no 9/12 (75%)	Yes 5/12 (41.7%), no 7/12 (58.3%)	Yes 9/12 (75%), no 3/12 (25%)	Yes 10/12 (83.3%), no 2/12 (16.7%)	Yes 2/12 (16.7%), no 10/12 (83.3%)	Yes 9/12 (75%), no 3/12 (25%)	Yes 9/12 (75%), no 3/12 (25%)

*FDA*, Food and Drug Administration.

∗ The link takes the user to a separate Spanish webpage.

† The link takes the user directly to the prescribing information.

**Table II:** English biologic website analysis

Biologic	English-specific link on biologic website homepage?	English-specific description of psoriasis?	Psoriasis subtypes mentioned in English?	Payment resources listed in English?	English-specific resources listed? (ie, information to contact English-speaking representatives)	Safety information listed in English? (ie, allergy information)	Visual aids present on website?	Testimonials present in English?	Mention of contraindications and special populations in English?	FDA information listed to report adverse medication events in English?
Cimzia	N/A	Yes	Yes	Yes	Yes	Yes	Yes	Yes	Yes	Yes
Cosentyx	N/A	Yes	Yes	Yes	Yes	Yes	Yes	Yes	Yes	Yes
Enbrel	N/A	Yes	Yes	Yes	Yes	Yes	Yes	Yes	Yes	Yes
Humira	N/A	Yes	Yes	Yes	Yes	Yes	Yes	Yes	Yes	Yes
Ilumya	N/A	Yes	Yes	Yes	Yes	Yes	Yes	Yes	Yes	Yes
Remicade	N/A	Yes	Yes	Yes	Yes	Yes	Yes	Yes	Yes	Yes
Siliq	N/A	Yes	Yes	Yes	Yes	Yes	Yes	Yes	Yes	Yes
Simponi	N/A	Yes	Yes	Yes	Yes	Yes	Yes	No	Yes	Yes
Skyrizi	N/A	Yes	Yes	Yes	Yes	Yes	Yes	Yes	Yes	Yes
Stelara	N/A	Yes	Yes	Yes	Yes	Yes	Yes	Yes	Yes	Yes
Taltz	N/A	Yes	Yes	Yes	Yes	Yes	Yes	Yes	Yes	Yes
Tremfya	N/A	Yes	Yes	Yes	Yes	Yes	Yes	Yes	Yes	Yes
Summary	N/A	Yes 12/12 (100%), no 0/12 (0%)	Yes 12/12 (100%), no 0/12 (0%)	Yes 12/12 (100%), no 0/12 (0%)	Yes 12/12 (100%), no 0/12 (0%)	Yes 12/12 (100%), no 0/12 (0%)	Yes 12/12 (100%), no 0/12 (0%)	Yes 11/12 (91.7%), no 1/12 (8.3%)	Yes 12/12 (100%), no 0/12 (0%)	Yes 12/12 (100%), no 0/12 (0%)

*FDA*, Food and Drug Administration.

The lack of information available to Spanish-speaking patients with psoriasis can contribute to undertreatment and poorer outcomes in LatinX individuals. Only 8 of the United States Food and Drug Administration-approved biologics for psoriasis had a homepage link to information in Spanish, with 3 of those websites directing users to the prescribing information in Spanish (Table I). Although the prescribing information links provide helpful information on medication adverse effects and indications, the information is presented in a way that is difficult to understand for most individuals, regardless of educational status.

Spanish homepage links were not consistently identified in one homepage location, and several supplemental materials could only be located externally using a major search engine. Two biologics also had either the homepage or supplementary links that were broken and not accessible.

Making access to resources more widely available may increase the availability of equitable health care for all patients. Improvements to psoriasis resources can include literacy levels, prescribing staff language skills, factors that influence clinicians’ bilingual counseling ability, and language assistance programs for medical professionals and patients.^3^ Empowering patients from diverse communities by ensuring linguistic equity may create a more inclusive health care system.

## Conflicts of interest

Dr Pichardo has worked on the advisory board for Novartis. Dr Feldman has received research, speaking, and/or consulting support from Galderma, GSK/Stiefel, Almirall, Leo Pharma, Boehringer Ingelheim, Mylan, Celgene, Pfizer, Valeant, AbbVie, Samsung, Janssen, Lilly, Menlo, Merck, Novartis, Regeneron, Sanofi, Novan, Qurient, National Biological Corporation, Caremark, Advance Medical, Sun Pharma, Suncare Research, Informa, UpToDate, and National Psoriasis Foundation. He is founder and majority owner of www.DrScore.com and founder and part owner of Causa Research, a company dedicated to enhancing patients’ adherence to treatment. Authors Oscherwitz, Lovell, and Markham have no conflicts of interest to declare.
